# Supplementary material for: DNA methylation-based high-resolution mapping of long-distance chromosomal interactions in nucleosome-depleted regions
Source: Nat Commun. 2024 May 22;15:4358. doi: 10.1038/s41467-024-48718-y (PMC11111806; doi:10.1038/s41467-024-48718-y)
Supplement: Supplementary file 1 — Supplementary Information [file 41467_2024_48718_MOESM1_ESM.pdf]

Supplementary Information

DNA Methylation-Based High-Resolution Mapping of Long-Distance Chromosomal Interactions in Nucleosome-Depleted Regions

Yi Li [1,2], James Lee [1,2], and Lu Bai [1,2,3*]

[1]*Department of Biochemistry and Molecular Biology,* [2]*Center for Eukaryotic Gene Regulation,*
[3]*Department of Physics, The Pennsylvania State University, University Park, PA, 16802, USA*
*Corresponding author: lub15@psu.edu, (814) 863-4824

Supplementary Information includes:

Supplementary Fig. 1: Parameters determination for the MTAC procedure.

Supplementary Fig. 2: Hi-C experiments for testing the effect of LacO insertion and DNA methylation on 3D genome conformation.

Supplementary Fig. 3: Additional MTAC data and comparison with 3C-based methods.

Supplementary Fig. 4: Examples of MTAC-captured interactions with YBP2 VP and 3C validation.

Supplementary Fig. 5: Mlp1 M.CviPI-ID and the comparison to Mlp1 ChIP-seq data.

Supplementary Fig. 6: MTAC data at YBP2 VP with mlp1/mlp2 double deletion.

Supplementary Fig. 7: Hi-C experiments.

Supplementary Fig. 8: MTAC during mating-type switch.

Supplementary Fig. 9: Venn diagram of the overlap of interactions made by different VPs.

Supplementary Table 1: Plasmid and strain list.

Supplementary Table 2: Primer list.

Supplementary Table 3: Genomic Dataset summary.

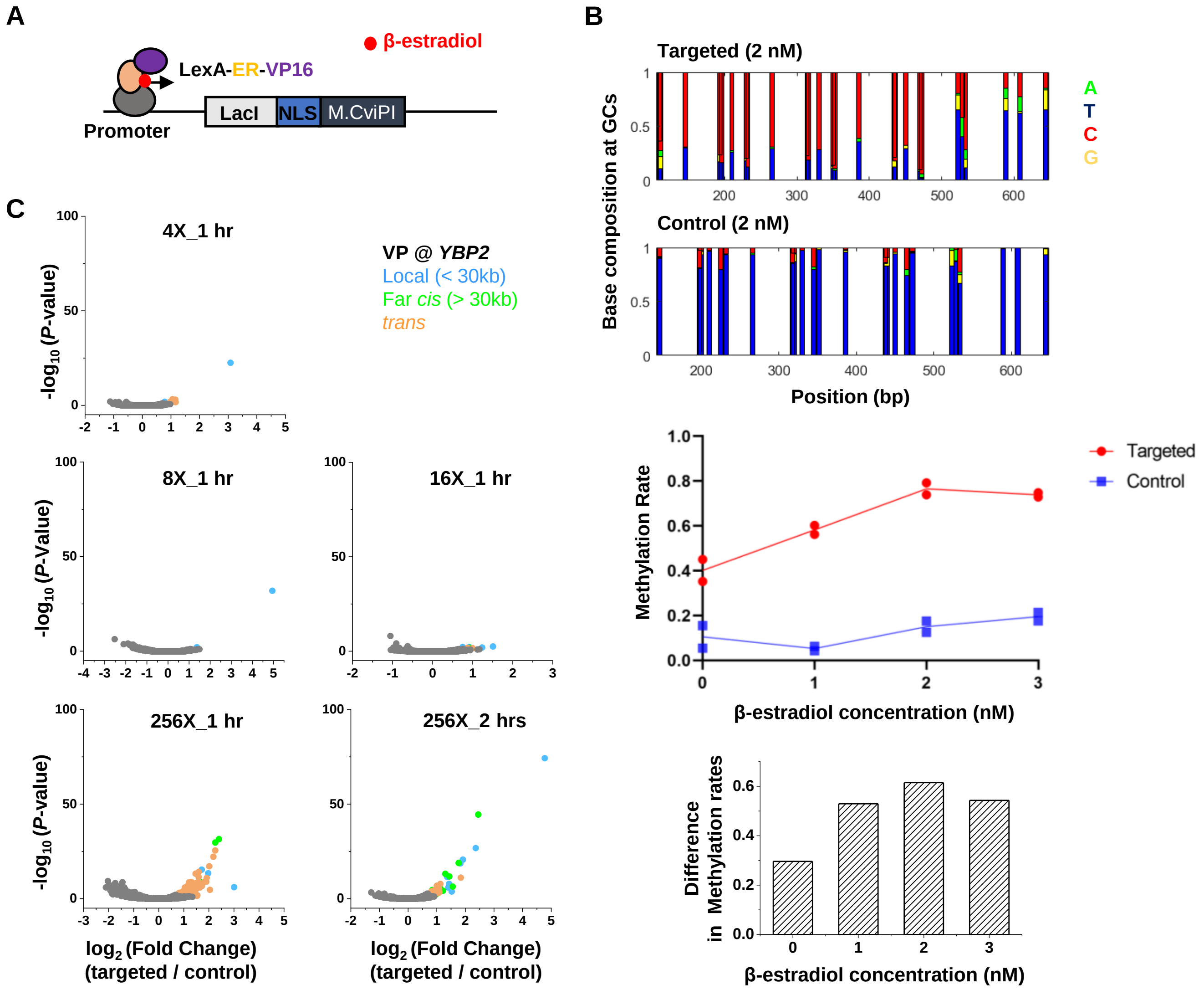

Supplementary Fig. 1. Parameters determination for the MTAC procedure. A) Inducible expression system of the LacI-M.CviPI fusion protein. The fusion protein with Nuclear Localization Sequence (NLS) is controlled by a constitutively expressed LexA-ER-VP16 artificial transcription factor, which is activated upon addition of β-estradiol. **B)** Optimizing the inducer concentrations by the local methylation rate. The local methylation is measured by Sanger sequencing of a bisulfite-converted PCR product near the *MATa* VP. Nucleotide composition is calculated for cytosine in each “GC” context, where the targeted strain has more methyl-C that is resistant to bisulfite conversion (top panel). Methylation rate is calculated as the average fraction of "C" at each “GC” position on this PCR product for both targeted (red) and control (blue) strains (Middle panel). The differences between the two are plotted in the bottom panel. **C)** Volcano plot of MTAC signals from the *YBP2* VP with different induction time and LacO copy numbers. 2 nM β-estradiol is used for all conditions. Statistical analysis (**C**) is based on two-sided Wald test with *P*-value adjustment for multiple comparisons (Benjamini & Hochberg method) by DESeq2 (n = 2 biologically independent samples per group).

Supplementary Fig. 2

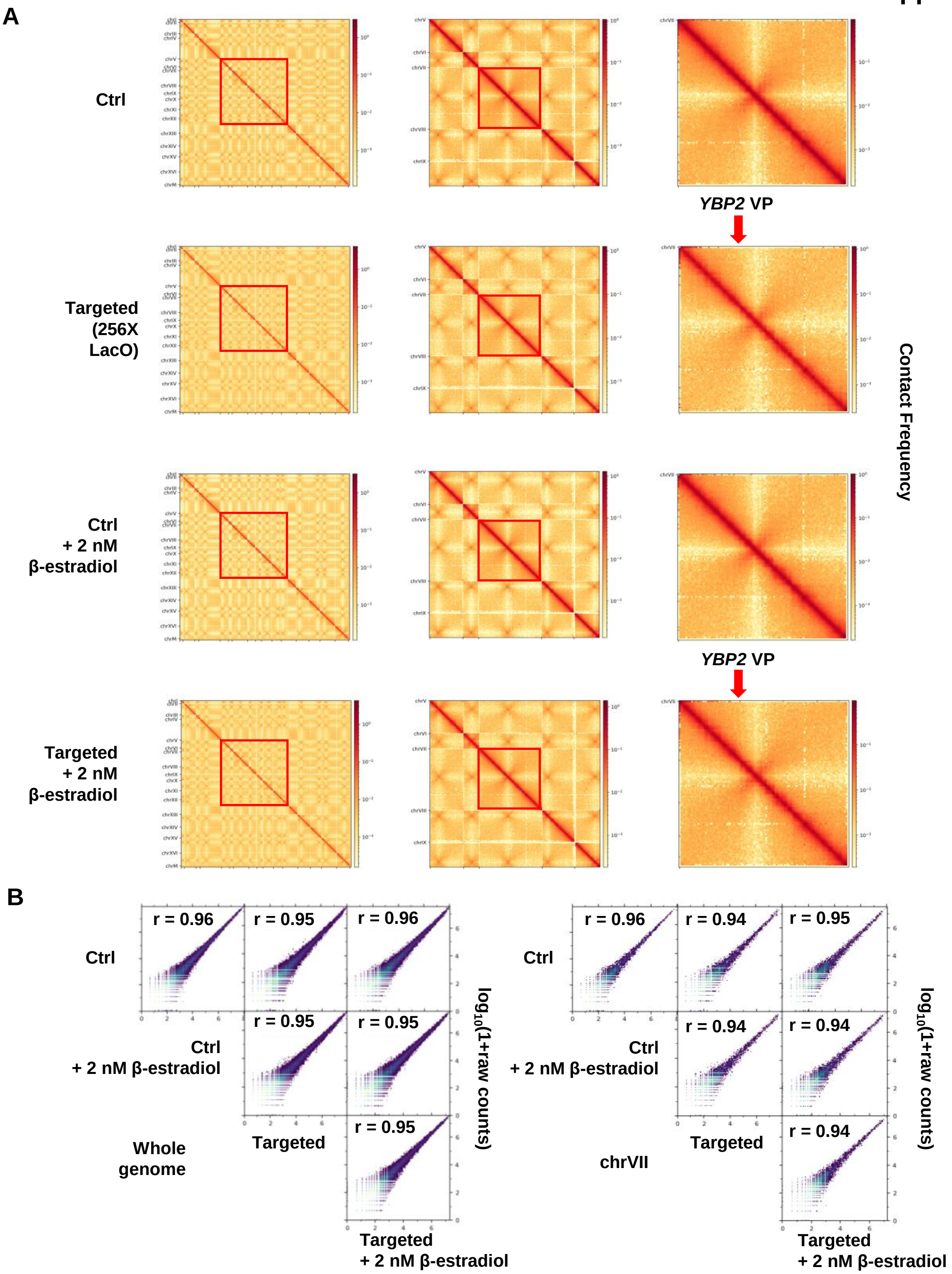

Supplementary Fig. 2. Hi-C experiments for testing the effect of LacO insertion and DNA methylation on 3D genome conformation. A) Hi-C matrices. Top to bottom: Control strain without β-estradiol, targeted strain at *YBP2* VP without β-estradiol, control strain with 2 nM β-estradiol, targeted strain with 2 nM β-estradiol. LacO insertion site was marked by red arrows. **B)** Pearson's correlation "*r*" between Hi-C contact frequencies. Left: whole genome, Right: only for chrVII, in which *YBP2* VP is inserted. The values of "r"s are comparable to typical correlation efficiency between biological replica of Hi-C using the same strain, indicating that LacO insertion and DNA methylation do not significantly impact 3D genome conformation.

Supplementary Fig. 3

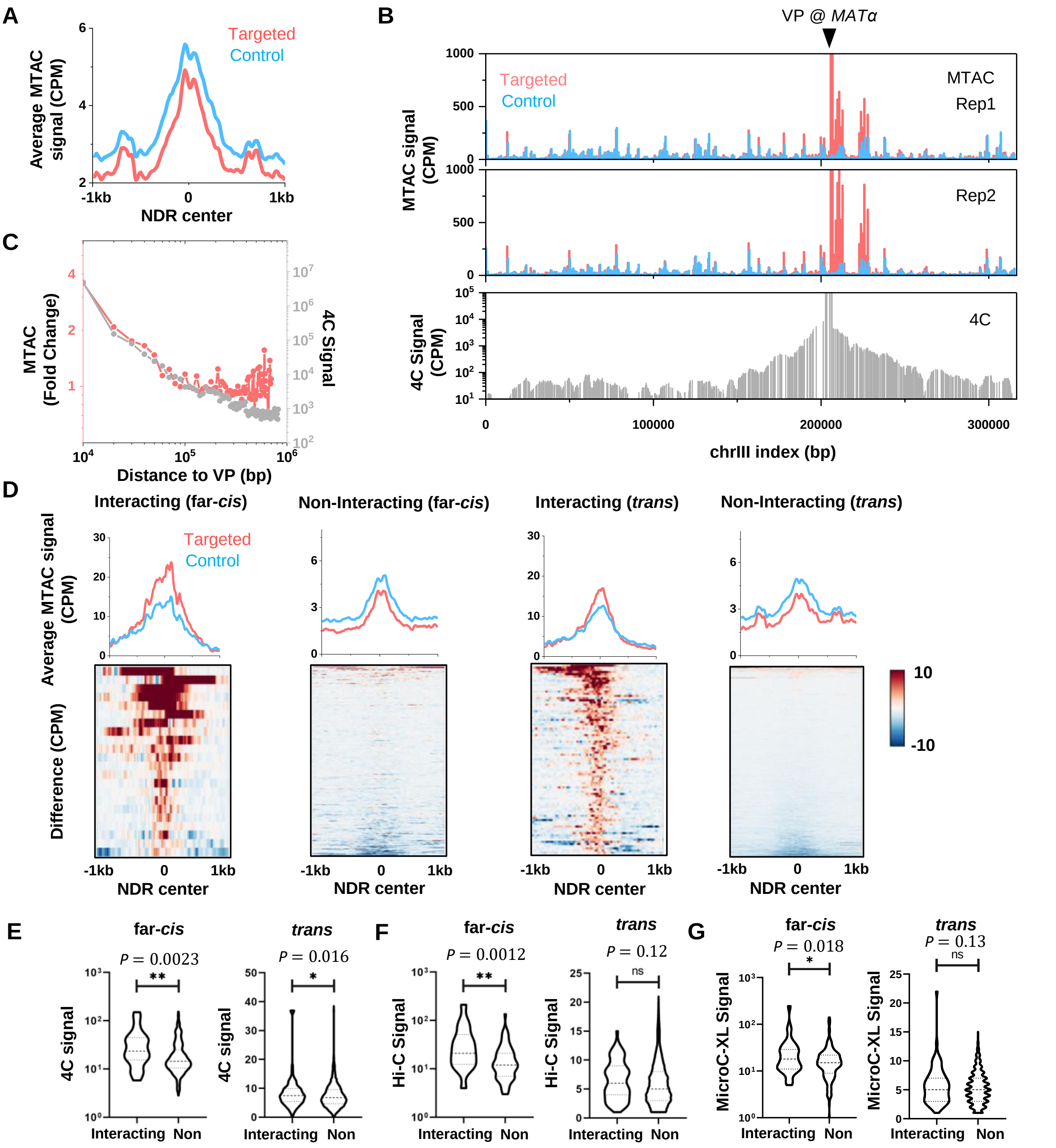

Supplementary Fig. 3. Additional MTAC data and comparison with 3C-based methods. A) Averaged MTAC signal from the *YBP2* VP as a function of distance to the genome-wide NDR centers. MTAC signals from the targeted and control strains are shown in pink and blue, respectively. **B)** MTAC and 4C signals from the *MATα* viewpoint. Two biological replica are shown for MTAC signal. **C)** Averaged local MTAC (pink) and 4C signals (gray) as a function of distance to the VP. The averaged data from three VPs (*YBP2*, *MATa,* and *MATα*) are shown. **D)** Averaged MTAC signals and heatmaps of far-*cis* and *trans* NDRs from the *YBP2* VP that are either interacting or non-interacting. **E-G)** 4C, Hi-C and Micro-C XL signals (5 kb resolution) near the captured interacting and non-interacting far-*cis* and *trans* NDRs of the *YBP2* VP. Statistical analysis is based on two-tailed students' t-test. Source data are provided as a Source Data file.

Supplementary Fig. 4

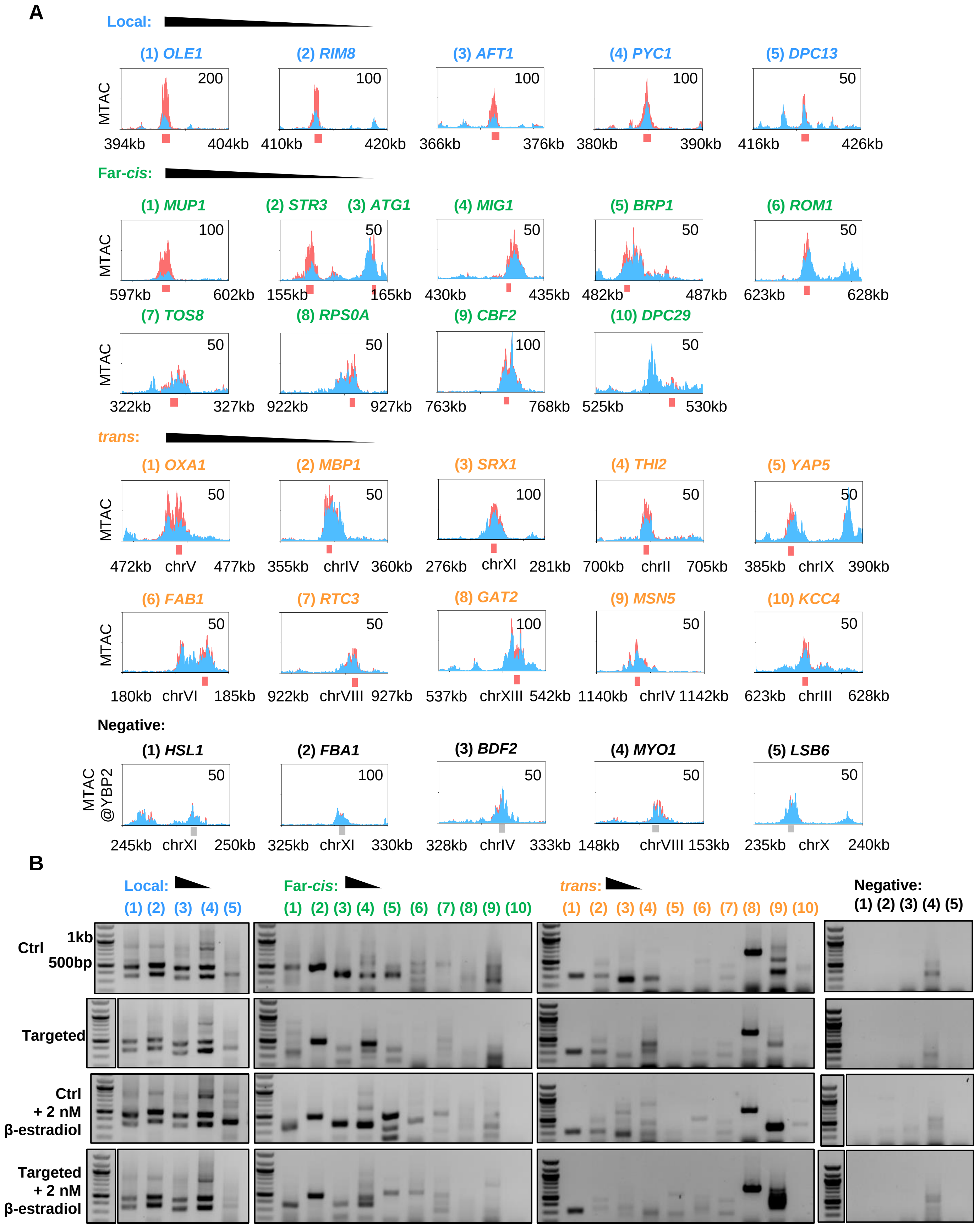

Supplementary Fig. 4. Examples of MTAC-captured interactions with YBP2 VP and 3C validation. A) Examples of different types of interactions with YBP2 VP that are ranked by the MTAC signals. Top to bottom: Local interactions (n = 5), Far-*cis* interactions (n = 10), *trans* interactions (n = 10), and regions with no interactions (n = 5). **B)** 3C validations for all the interacting sites shown in A. 3C was performed in four different conditions to test the effect from LacO array insertion and DNA methylation. Top to bottom: control strain without β-estradiol, targeted strain at *YBP2* VP without β-estradiol, control strain with 2 nM β-estradiol, targeted strain with 2 nM β-estradiol. Two pairs of primers were used for each interaction to test two conformations of interaction, the one with the strongest intensity was shown. Same molecular weight maker is used for all the plots. The experiments were repeated twice with similar results. Uncropped scans are provided in the Source Data file.

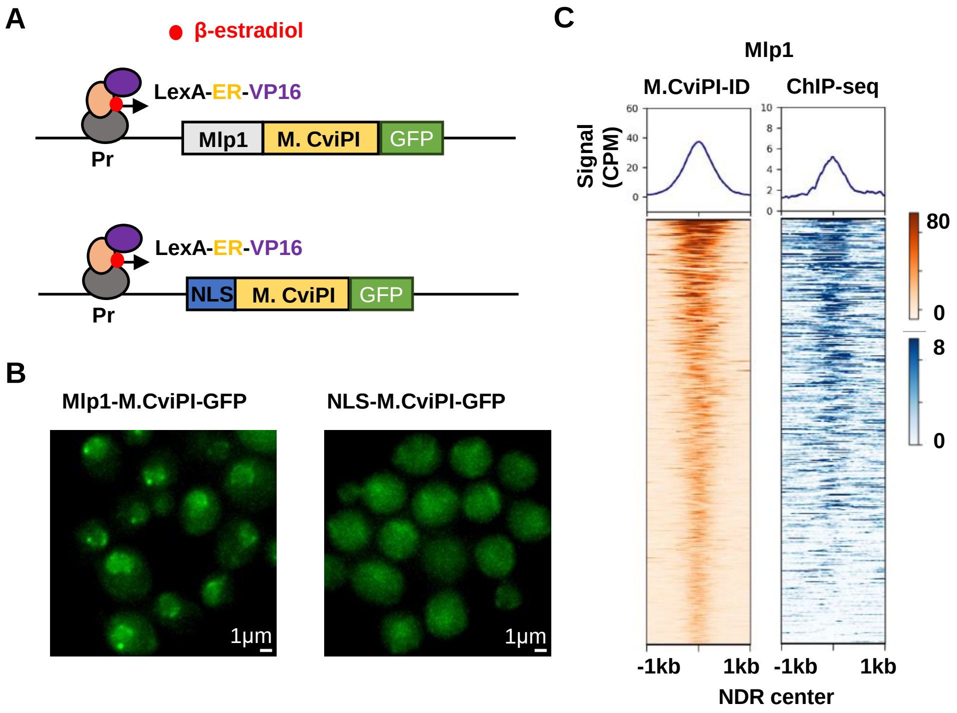

Supplementary Fig. 5. Mlp1 M.CviPI-ID and the comparison to Mlp1 ChIP-seq data. A) Inducible expression of the Mlp1-M.CviPI fusion protein. The fusion protein and the free M.CviPI control are regulated by LexA-ER-VP16, which is activated upon addition of β-estradiol. **B)** Imaging of the fusion proteins in live yeast cells. Mlp1-M.CviPI-GFP shows a nuclear envelope localization while NLS-M.CviPI-GFP shows a diffusive localization inside the nucleus upon induction with 10 nM β-estradiol. **C)** Comparison between Mlp1-M.CviPI ID signal and published Mlp1 ChIP-seq signal (Forey *et al*.[33]). Mlp1 M.CviPI-ID signal is normalized by subtracting the free-M.CviPI signal. Mlp1 ChIP-seq signal is normalized by subtracting the free-GFP ChIP-seq signal.

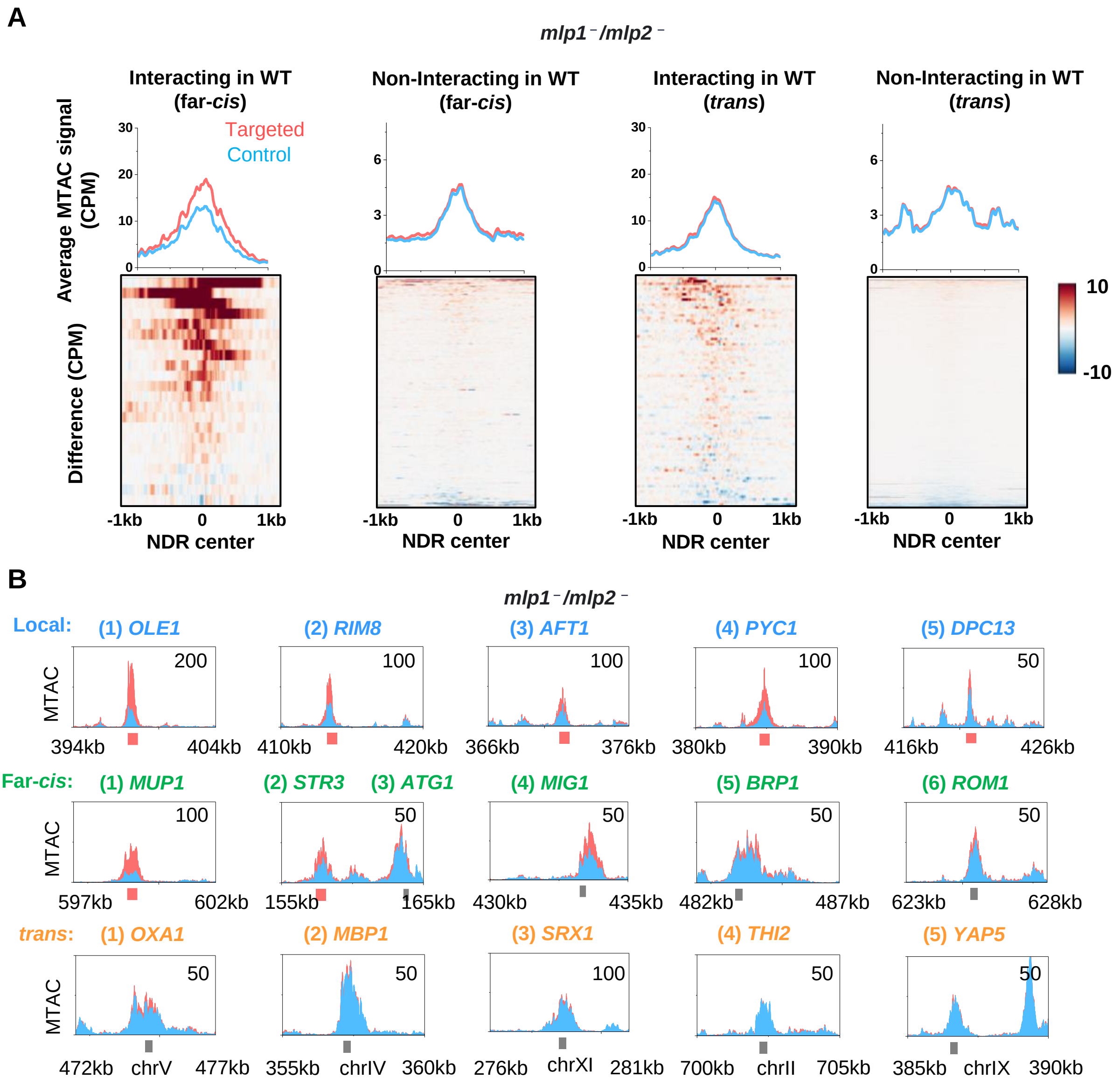

Supplementary Fig. 6. MTAC data at *YBP2* VP with *mlp1/mlp2* double deletion. A) Averaged MTAC signals and heatmaps of the *mlp1⁻/mlp2⁻* mutant at NDRs that show far-*cis* and *trans*-interactions with the VP in WT. Corresponding data of the WT are shown in Supplementary Fig. 3D. **B)** Example tracks of MTAC data in the *mlp1⁻/mlp2⁻* mutant at NDRs that show local, far-*cis* and *trans*-interaction with the VP in WT. Corresponding data of the WT are shown in Supplementary Fig. 4A.

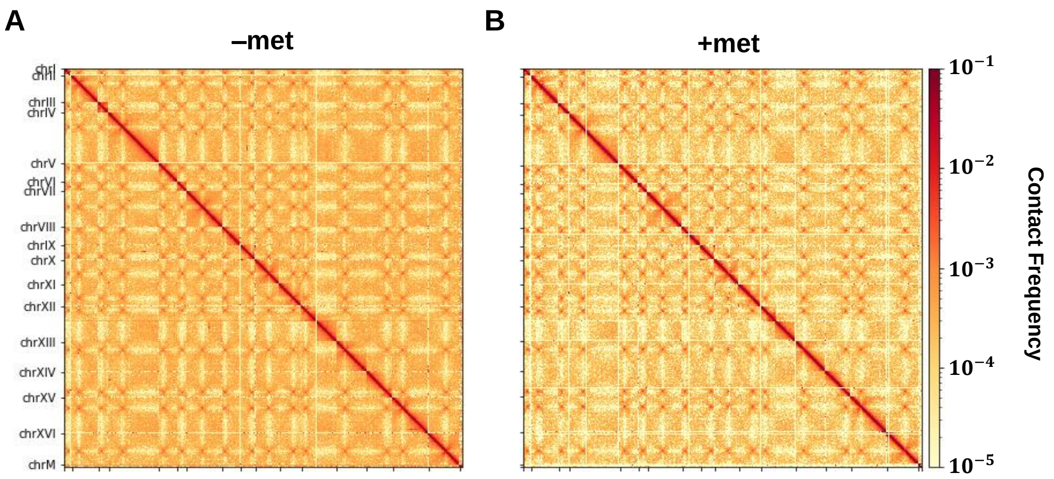

Supplementary Fig. 7. Hi-C experiments. A) Hi-C data measured in this study in −met condition. **B)** Hi-C data in +met condition[35].

Supplementary Fig. 8

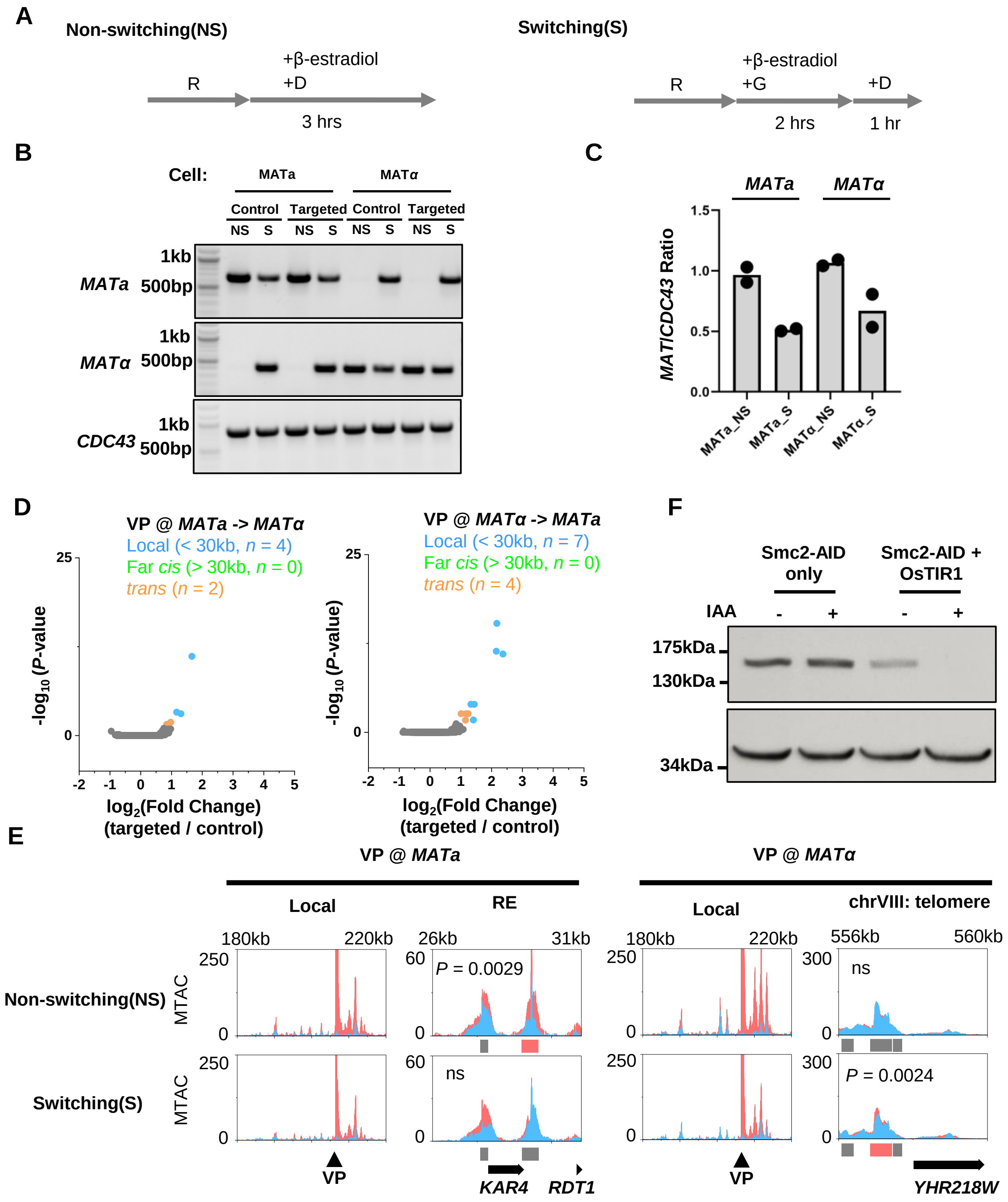

Supplementary Fig. 8. MTAC during mating-type switch. A) Experimental procedure of the mating-type switch assay in Non-switching (NS) and Switching conditions (S). R: raffinose, D: glucose, and G: galactose. **B)** Mating-type switch efficiency. MAT genes before and after switch are quantified by PCR using *MATa* or *MATα* – specific primers. *CDC43* gene is used as the control for total amount of genomic DNA. The experiments were repeated twice with similar results. **C)** Quantification of *MAT* gene before and after switch. The ratio of *MAT* gene and *CDC43* PCR product is calculated for each condition (n = 2 biological replicates). **D)** Volcano plot of MTAC signals from the *MAT* VP in switching *MATa* and *MATα* cells. **E)** Representative tracks of MTAC data near the VP and interacting NDRs. **F)** Western blot of V5-tagged Smc2 ± IAA in cells with or without Tir1. Actin is used as input loading control. The experiments were repeated twice with similar results. Statistical analysis (**D**) is based on two-sided Wald test with P-value adjustment for multiple comparisons (Benjamini & Hochberg method) by DESeq2 (n = 2 biologically independent samples per group).

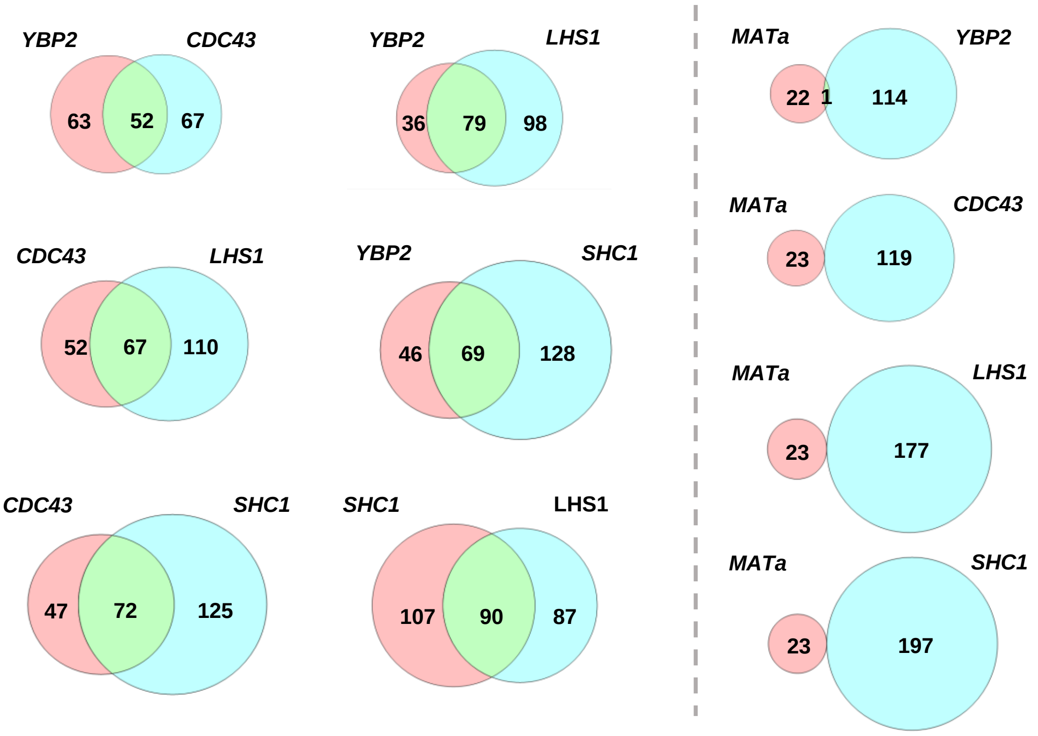

Supplementary Fig. 9. Venn diagram of the overlap of interactions made by different VPs. Interactions made by most VPs show significant overlaps (left). Interactions made by *MATa* VP have little or no overlap with other VPs (right).

Supplementary Table 1: Plasmid and strain list

Plasmid	Note	Note
pLB51c	2xlexO-GAL1corepr-LacI-SV40NLS-M.CviPI and ADH1pr-LexA-ERHBD-VP16AD with LEU2 marker	
pLB52a-4X	4X LacO with HIS3 marker	
pYL1a-8X	8X LacO with HIS3 marker	
pYL1a-16X	16X LacO with HIS3 marker	
pSR15-YBP2	256X LacO with YBP2 homologs and HIS3 marker	
pSR15-OLE1	256X LacO with OLE1 homologs and HIS3 marker	
pSR15-PUS2	256X LacO with PUS2 homologs and HIS3 marker	
pSR15-MNR2	256X LacO with MNR2 homologs and HIS3 marker	
pSR15-PCL10	256X LacO with PCL10 homologs and HIS3 marker	
pSR15-MBR1	256X LacO with MBR1 homologs and HIS3 marker	
pSR15-VHR1	256X LacO with VHR1 homologs and HIS3 marker	
pSR15-SHC1	256X LacO with SHC1 homologs and HIS3 marker	
pSR15-LHS1	256X LacO with LHS1 homologs and HIS3 marker	
pSR15-LSM4	256X LacO with LSM4 homologs and HIS3 marker	
pSR15-CDC43	256X LacO with CDC43 homologs and HIS3 marker	
pSR15-MET6	256X LacO with TRP2 homologs and HIS3 marker	
pSR15-MET13	256X LacO with MET13 homologs and HIS3 marker	
pSR15-TAF2	256X LacO with TAF2 homologs and HIS3 marker	
pYL07	2xlexO-GAL1corepr-Mlp1-M.CviPI and ADH1pr-LexA-ERHBD-VP16AD with LEU2 marker	
pYL09	2xlexO-GAL1corepr-SV40NLS-M.CviPI and ADH1pr-LexA-ERHBD-VP16AD with LEU2 marker	
pYL15	2xlexO-GAL1corepr-Mlp1-M.CviPI-GFP and ADH1pr-LexA-ERHBD-VP16AD with LEU2 marker	
pYL16	2xlexO-GAL1corepr-SV40NLS-M.CviPI-GFP and ADH1pr-LexA-ERHBD-VP16AD with LEU2 marker	

Strain	Genotype	Note
yLB121a	*MATa, his3-11,15, leu2-3,112, trp1-1, ura3-1, can1-100, ade2-1, ADH1::2xlexO-GAL1corepr-LacI-SV40NLS-M.CviPI:ADH1pr-LexA-ERHBD-VP16AD::LEU2*	background
yLB121a-4X	*MATa, his3-11,15, leu2-3,112, trp1-1, ura3-1, can1-100, ade2-1, ADH1::2xlexO-GAL1corepr-LacI-SV40NLS-M.CviPI:ADH1pr-LexA-ERHBD-VP16AD::LEU2, YBP2::4XLacO::HIS3*	YBP2 VP, 4X
yLB121a-8X	*MATa, his3-11,15, leu2-3,112, trp1-1, ura3-1, can1-100, ade2-1, ADH1::2xlexO-GAL1corepr-LacI-SV40NLS-M.CviPI:ADH1pr-LexA-ERHBD-VP16AD::LEU2, YBP2::8XLacO::HIS3*	YBP2 VP, 8X
yLB121a-16X	*MATa, his3-11,15, leu2-3,112, trp1-1, ura3-1, can1-100, ade2-1, ADH1::2xlexO-GAL1corepr-LacI-SV40NLS-M.CviPI:ADH1pr-LexA-ERHBD-VP16AD::LEU2, YBP2::16XLacO::HIS3*	YBP2 VP, 16X
yLB121a-YBP2	*MATa, his3-11,15, leu2-3,112, trp1-1, ura3-1, can1-100, ade2-1, ADH1::2xlexO-GAL1corepr-LacI-SV40NLS-M.CviPI:ADH1pr-LexA-ERHBD-VP16AD::LEU2, YBP2::256XLacO::HIS3*	YBP2 VP
yLB121a-OLE1	*MATa, his3-11,15, leu2-3,112, trp1-1, ura3-1, can1-100, ade2-1, ADH1::2xlexO-GAL1corepr-LacI-SV40NLS-M.CviPI:ADH1pr-LexA-ERHBD-VP16AD::LEU2, OLE1::256XLacO::HIS3*	OLE1 VP
yLB121a-PUS2	*MATa, his3-11,15, leu2-3,112, trp1-1, ura3-1, can1-100, ade2-1, ADH1::2xlexO-GAL1corepr-LacI-SV40NLS-M.CviPI:ADH1pr-LexA-ERHBD-VP16AD::LEU2, PUS2::256XLacO::HIS3*	PUS2 VP
yLB121a-MNR2	*MATa, his3-11,15, leu2-3,112, trp1-1, ura3-1, can1-100, ade2-1, ADH1::2xlexO-GAL1corepr-LacI-SV40NLS-M.CviPI:ADH1pr-LexA-ERHBD-VP16AD::LEU2, MNR2::256XLacO::HIS3*	MNR2 VP
yLB121a-PCL10	*MATa, his3-11,15, leu2-3,112, trp1-1, ura3-1, can1-100, ade2-1, ADH1::2xlexO-GAL1corepr-LacI-SV40NLS-M.CviPI:ADH1pr-LexA-ERHBD-VP16AD::LEU2, PCL10::256XLacO::HIS3*	PCL10 VP
yLB121a-MBR1	*MATa, his3-11,15, leu2-3,112, trp1-1, ura3-1, can1-100, ade2-1, ADH1::2xlexO-GAL1corepr-LacI-SV40NLS-M.CviPI:ADH1pr-LexA-ERHBD-VP16AD::LEU2, MBR1::256XLacO::HIS3*	MBR1 VP
yLB121a-VHR1	*MATa, his3-11,15, leu2-3,112, trp1-1, ura3-1, can1-100, ade2-1, ADH1::2xlexO-GAL1corepr-LacI-SV40NLS-M.CviPI:ADH1pr-LexA-ERHBD-VP16AD::LEU2, VHR1::256XLacO::HIS3*	VHR1 VP
yLB121a-SHC1	*MATa, his3-11,15, leu2-3,112, trp1-1, ura3-1, can1-100, ade2-1, ADH1::2xlexO-GAL1corepr-LacI-SV40NLS-M.CviPI:ADH1pr-LexA-ERHBD-VP16AD::LEU2, SHC1::256XLacO::HIS3*	SHC1 VP
yLB121a-LHS1	*MATa, his3-11,15, leu2-3,112, trp1-1, ura3-1, can1-100, ade2-1, ADH1::2xlexO-GAL1corepr-LacI-SV40NLS-M.CviPI:ADH1pr-LexA-ERHBD-VP16AD::LEU2, LHS1::256XLacO::HIS3*	LHS1 VP
yLB121a-LSM4	*MATa, his3-11,15, leu2-3,112, trp1-1, ura3-1, can1-100, ade2-1, ADH1::2xlexO-GAL1corepr-LacI-SV40NLS-M.CviPI:ADH1pr-LexA-ERHBD-VP16AD::LEU2, LSM4::256XLacO::HIS3*	LSM4 VP
yLB121a-CDC43	*MATa, his3-11,15, leu2-3,112, trp1-1, ura3-1, can1-100, ade2-1, ADH1::2xlexO-GAL1corepr-LacI-SV40NLS-M.CviPI:ADH1pr-LexA-ERHBD-VP16AD::LEU2, CDC43::256XLacO::HIS3*	CDC43 VP
yLB121a-TRP2	*MATa, his3-11,15, leu2-3,112, trp1-1, ura3-1, can1-100, ade2-1, ADH1::2xlexO-GAL1corepr-LacI-SV40NLS-M.CviPI:ADH1pr-LexA-ERHBD-VP16AD::LEU2, TRP2::256XLacO::HIS3*	TRP2 VP
yLB121a-MET13	*MATa, his3-11,15, leu2-3,112, trp1-1, ura3-1, can1-100, ade2-1, ADH1::2xlexO-GAL1corepr-LacI-SV40NLS-M.CviPI:ADH1pr-LexA-ERHBD-VP16AD::LEU2, MET13::256XLacO::HIS3*	MET13 VP
yLB121a-MATa	*MATa, his3-11,15, leu2-3,112, trp1-1, ura3-1, can1-100, ade2-1, ADH1::2xlexO-GAL1corepr-LacI-SV40NLS-M.CviPI:ADH1pr-LexA-ERHBD-VP16AD::LEU2, TAF2::256XLacO::HIS3*	MATa VP
yLB121α-MATα	*MATα, his3-11,15, leu2-3,112, trp1-1, ura3-1, can1-100, ade2-1, ADH1::2xlexO-GAL1corepr-LacI-SV40NLS-M.CviPI:ADH1pr-LexA-ERHBD-VP16AD::LEU2, TAF2::256XLacO::HIS3*	MATα VP
yLB121a-sw	*MATa, his3-11,15, leu2-3,112, trp1-1, ura3-1, can1-100, ade2-1, ADH1::2xlexO-GAL1corepr-LacI-SV40NLS-M.CviPI:ADH1pr-LexA-ERHBD-VP16AD::LEU2, GAL10pr::GAL10pr-HO::URA3*	Mating type switch
yLB121a-MATa-sw	*MATa, his3-11,15, leu2-3,112, trp1-1, ura3-1, can1-100, ade2-1, ADH1::2xlexO-GAL1corepr-LacI-SV40NLS-M.CviPI:ADH1pr-LexA-ERHBD-VP16AD::LEU2, TAF2::256XLacO::HIS3, GAL10pr::GAL10pr-HO::URA3*	Mating type switch
yLB121α-sw	*MATα, his3-11,15, leu2-3,112, trp1-1, ura3-1, can1-100, ade2-1, ADH1::2xlexO-GAL1corepr-LacI-SV40NLS-M.CviPI:ADH1pr-LexA-ERHBD-VP16AD::LEU2, GAL10pr::GAL10pr-HO::URA3*	Mating type switch
yLB121α-MATα-sw	*MATα, his3-11,15, leu2-3,112, trp1-1, ura3-1, can1-100, ade2-1, ADH1::2xlexO-GAL1corepr-LacI-SV40NLS-M.CviPI:ADH1pr-LexA-ERHBD-VP16AD::LEU2, TAF2::256XLacO::HIS3, GAL10pr::GAL10pr-HO::URA3*	Mating type switch
yLB121a-RE	*MATa, his3-11,15, leu2-3,112, trp1-1, ura3-1, can1-100, ade2-1, ADH1::2xlexO-GAL1corepr-LacI-SV40NLS-M.CviPI:ADH1pr-LexA-ERHBD-VP16AD::LEU2, re::KanMX*	RE depletion
yLB121a-MATa-RE	*MATa, his3-11,15, leu2-3,112, trp1-1, ura3-1, can1-100, ade2-1, ADH1::2xlexO-GAL1corepr-LacI-SV40NLS-M.CviPI:ADH1pr-LexA-ERHBD-VP16AD::LEU2, TAF2::256XLacO::HIS3, re::KanMX*	RE depletion
yLB121a-SIR2	*MATa, his3-11,15, leu2-3,112, trp1-1, ura3-1, can1-100, ade2-1, ADH1::2xlexO-GAL1corepr-LacI-SV40NLS-M.CviPI:ADH1pr-LexA-ERHBD-VP16AD::LEU2, sir2::KanMX*	SIR2 depletion
yLB121a-MATa-SIR2	*MATa, his3-11,15, leu2-3,112, trp1-1, ura3-1, can1-100, ade2-1, ADH1::2xlexO-GAL1corepr-LacI-SV40NLS-M.CviPI:ADH1pr-LexA-ERHBD-VP16AD::LEU2, TAF2::256XLacO::HIS3, sir2::KanMX*	SIR2 depletion
yLB121a-SMC2Tag	*MATa, his3-11,15, leu2-3,112, trp1-1, ura3-1, can1-100, ade2-1, ADH1::2xlexO-GAL1corepr-LacI-SV40NLS-M.CviPI:ADH1pr-LexA-ERHBD-VP16AD::LEU2, smc2::SMC2-IAA7::KanMX*	SMC2 tag
yLB121a-MATa-SMC2Ta	*MATa, his3-11,15, leu2-3,112, trp1-1, ura3-1, can1-100, ade2-1, ADH1::2xlexO-GAL1corepr-LacI-SV40NLS-M.CviPI:ADH1pr-LexA-ERHBD-VP16AD::LEU2, TAF2::256XLacO::HIS3, smc2::SMC2-IAA7::KanMX*	SMC2 tag
yLB121a-SMC2AID	*MATa, his3-11,15, leu2-3,112, trp1-1, ura3-1, can1-100, ade2-1, ADH1::2xlexO-GAL1corepr-LacI-SV40NLS-M.CviPI:ADH1pr-LexA-ERHBD-VP16AD::LEU2, smc2::SMC2-IAA7::KanMX, ho::OsTIR1::URA3*	SMC2 AID
yLB121a-MATa-SMC2AII	*MATa, his3-11,15, leu2-3,112, trp1-1, ura3-1, can1-100, ade2-1, ADH1::2xlexO-GAL1corepr-LacI-SV40NLS-M.CviPI:ADH1pr-LexA-ERHBD-VP16AD::LEU2, TAF2::256XLacO::HIS3, smc2::SMC2-IAA7::KanMX, ho::OsTIR1::URA3*	SMC2 AID
yYL38a	*MATa, his3-11,15, leu2-3,112, trp1-1, ura3-1, can1-100, ade2-1, ADH1::2xlexO-GAL1corepr-Mlp1-M.CviPI:ADH1pr-LexA-ERHBD-VP16AD::LEU2*	M. CviPI-ID
yYL41a	*MATa, his3-11,15, leu2-3,112, trp1-1, ura3-1, can1-100, ade2-1, ADH1::2xlexO-GAL1corepr-SV40NLS-M.CviPI:ADH1pr-LexA-ERHBD-VP16AD::LEU2*	M. CviPI-ID
yYL55a	*MATa, his3-11,15, leu2-3,112, trp1-1, ura3-1, can1-100, ade2-1, ADH1::2xlexO-GAL1corepr-Mlp1-M.CviPI-GFP:ADH1pr-LexA-ERHBD-VP16AD::LEU2*	M. CviPI-ID, imaging
yYL56a	*MATa, his3-11,15, leu2-3,112, trp1-1, ura3-1, can1-100, ade2-1, ADH1::2xlexO-GAL1corepr-SV40NLS-M.CviPI-GFP:ADH1pr-LexA-ERHBD-VP16AD::LEU2*	M. CviPI-ID, imaging
yMD291	*MATa, his3-11,15, leu2-3,112, trp1-1, ura3-1, can1-100, ade2-1, tor1-1, fpr::NAT, bar1, HIS3::REV1pr-tetR-mCherry-REV1pr-LacI-GFP, ADE2::REV1pr-LacI-REV1pr-tetR, Chr15::497047_LacO, Chr15::834487_TetO*	Hi-C
yJL60a	*MATa, his3-11,15, leu2-3,112, trp1-1, ura3-1, can1-100, ade2-1, MET4::MET4-TAP::HIS3*	MET4 ChIP
yJL59a	*MATa, his3-11,15, leu2-3,112, trp1-1, ura3-1, can1-100, ade2-1, CDC20::MET3pr-mCherry::URA3, CWC23::MET3pr-GFP::KanMX*	RNA-seq
yYL79a	*MATa, his3-11,15, leu2-3,112, trp1-1, ura3-1, can1-100, ade2-1, ADH1::2xlexO-GAL1corepr-LacI-SV40NLS-M.CviPI:ADH1pr-LexA-ERHBD-VP16AD::LEU2, MLP2::HygoMX, MLP1::KanMX,*	background, MLP1/2 deletion
yYL79a-YBP2	*MATa, his3-11,15, leu2-3,112, trp1-1, ura3-1, can1-100, ade2-1, ADH1::2xlexO-GAL1corepr-LacI-SV40NLS-M.CviPI:ADH1pr-LexA-ERHBD-VP16AD::LEU2, MLP2::HygoMX, MLP1::KanMX, YBP2::256XLacO::HIS3*	YBP2 VP, MLP1/2 deletion

GAL1corepr, *GAL1* core promoter; *SV40NLS*, Simian Virus 40 nuclear localization signal; *ERHBD*, human estrogen receptor alpha hormone binding domain; *VP16AD*, VP16 activation domain

Supplementary Table 2: Primer list

Primer	Sequence	Note
QPF-RE	TAGGAAGTTTACACGATGTTCTCGC	qPCR primer for enrichment at RE
QPR-RE	CGAATAAGTACCTCGACTTCAAATC	qPCR primer for enrichment at RE
QPF-Tel_12	TATTACCCTGCCGAATAAGAAAACAG	qPCR primer for enrichment at Tel12
QPR-Tel_12	GGCAATATTTCCTGTTTAGGCGATTG	qPCR primer for enrichment at Tel12
QPF-N	TTCTGATCGTATATCTCTCAGGG	qPCR primer for enrichment at negative ctrl
QPR-N	GTTTCGTGTCGCTTAATCTATG	qPCR primer for enrichment at negative ctrl
PFm1-LacO_MAT	TTGAGGGATTTTTGGGTATTTGAG	Bisulfite sanger sequencing primer for local methylation
PRm1-LacO_MAT	CTATCCTATTTAAAACTCCCCATCATC	Bisulfite sanger sequencing primer for local methylation
PF-MAT-a	AATCATCAATATCACCCCAAGCACGG	Mating type switch efficiency
PF-MAT-alpha	GCACGGAATATGGGACTACTTCG	Mating type switch efficiency
PR-MAT	AGTCACATCAAGATCGTTTATGG	Mating type switch efficiency
PF-CDC43	GATGCTGATTTATATCACAGCTGTCTAG	Mating type switch efficiency
PR-CDC43	AGATGTTCAAATCTATGCAGCCAGC	Mating type switch efficiency
PF-4C_YBP2	AGTACCAAGAGTAAATCACCTTGCG	4C at YBP2
PR-4C_YBP2	ACACTATGTTTTGAGTCATCCAAACTTTC	4C at YBP2
PF-4C_MAT	TTGTGAAGCCGAAGGTAACTAGCAAAC	4C at MAT
PR-4C_MAT	CTGTTGATTAGGGTGTACATCACCC	4C at MAT
PF1-3C_YBP2	TGCAATGAATCTAATAACGACACTAAG	3C
PR1-3C_YBP2	GTAGTATTGGTGGCATATCTGTAAAACTG	3C
PF1-3C_OLE1	TCAGACACACCTATCCCTATTGTTAC	3C
PF1-3C_RIM8	AAGAGTCATGGTATTGTTGGCAGTTAC	3C
PF1-3C_AFT1	CATAGAACATGCGTCACCGATTAATTC	3C
PF1-3C_PYC1	AATCTCTACTTGCAACAAGAGTCAG	3C
PR1-3C_DPC13	TTATACACGGAAGGCGCTAGTAAGAC	3C
PF1-3C_MUP1	GCATCTTACAATGTATGCCACGATACC	3C
PR1-3C_STR3	AACAACTGGCGCATACGTAGCTATATT	3C
PF1-3C_ATG1	TGACACTAAGTAACGAAAGATATCTGTAAAC	3C
PR1-3C_MIG1	GAAGGTTGTGGGCTCTCCAATAAATAG	3C
PF1-3C_BRP1	TATTCCTGTCAAAAGCATGTCAGAGG	3C
PR1-3C_ROM1	TTAGTTCGCTATCTAGCAGCGGTGG	3C
PF1-3C_TOS8	GAGCATATCTCAACTCCCAATTTCTATC	3C
PF1-3C_RPS0A	CCACGTTAAAAGCTCGAATTCTTCGC	3C
PR1-3C_CBF2	AGCTGCCGTTCAATGTTTGTACTTCG	3C
PF1-3C_DPC29	TTTAGTACGCACGTACGCAAGTTTCAG	3C
PR1-3C_OXA1	ATGCCGACACTATAGCATACATGTG	3C
PR1-3C_MBP1	TGTGAGTTAGGAAAACGAACTGTAATG	3C
PF1-3C_SRX1	AGTCATTACAGTAGCCCTGTTGGGTAAG	3C
PR1-3C_THI2	ATGCAGATTCAAGAAACGCAGATG	3C
PF1-3C_YAP5	TTAGTTTCCGACGAATTGAGCTGAAGC	3C
PR1-3C_FAB1	TATTTCAGGTGCATAGGGTAACTGG	3C
PR1-3C_RTC3	AATATTCGTCTACAAGCTCTTCGGATGC	3C
PF1-3C_GAT2	CCTTTGCTCTAATATTGTTTTCGTTCATAG	3C
PF1-3C_MSN5	CAGAGGTCATTGTAAGAAATGACGTG	3C
PR1-3C_KCC4	CTAACCCCTTATTTCCTCATTTCCTC	3C
PR1-3C_HSL1	ACGTGGCTCGTTTTTGAAACGTGAC	3C
PF1-3C_FBA1	TTATTCATTTCTTAGGGCTTGCGTCAGC	3C
PF1-3C_BDF2	TTGTCTCCCATTTCCATTAGTAATGC	3C
PF1-3C_MYO1	TCACAGGGTAATGCTGAGATAGCTTTTC	3C
PR1-3C_LSB6	TCAGTGAGCCGACTCTACTACAATGC	3C

All primers are purchased from Integrated DNA Technologies

Supplementary Table 3: Genomic Dataset summary

Type	Name	Note	# of uniquely mapped reads	accession number	Source
MTAC	Ctrl_1_1hr	Ctrl, MeDIP, 1hr, 0X, rep1	2563387	GSM7762063	This study
	Ctrl_2_1hr	Ctrl, MeDIP, 1hr, 0X, rep2	2609825	GSM7762064	This study
	YBP2_1_1hr_4X	YBP2, MeDIP, 1hr, 4X, rep1	1912850	GSM7762065	This study
	YBP2_2_1hr_4X	YBP2, MeDIP, 1hr, 4X, rep2	1897716	GSM7762066	This study
	YBP2_1_1hr_8X	YBP2, MeDIP, 1hr, 8X, rep1	1071673	GSM7762067	This study
	YBP2_2_1hr_8X	YBP2, MeDIP, 1hr, 8X, rep2	1882979	GSM7762068	This study
	YBP2_1_1hr_16X	YBP2, MeDIP, 1hr, 16X, rep1	2876922	GSM7762069	This study
	YBP2_2_1hr_16X	YBP2, MeDIP, 1hr, 16X, rep2	1744597	GSM7762070	This study
	YBP2_1_1hr_256X	YBP2, MeDIP, 1hr, 256X, rep1	7028231	GSM7762071	This study
	YBP2_2_1hr_256X	YBP2, MeDIP, 1hr, 256X, rep2	5777646	GSM7762072	This study
	Ctrl_1	Ctrl, MeDIP, 2hr, 0X, rep1	7576467	GSM7762073	This study
	Ctrl_2	Ctrl, MeDIP, 2hr, 0X, rep2	6497995	GSM7762074	This study
	YBP2_1	YBP2, MeDIP, 2hr, 256X, rep1	2750440	GSM7762075	This study
	YBP2_2	YBP2, MeDIP, 2hr, 256X, rep2	2836266	GSM7762076	This study
	PUS2_1	PUS2, MeDIP, 2hr, 256X, rep1	2771614	GSM7762077	This study
	PUS2_2	PUS2, MeDIP, 2hr, 256X, rep2	4293136	GSM7762078	This study
	OLE1_1	OLE1, MeDIP, 2hr, 256X, rep1	2589806	GSM7762079	This study
	OLE1_2	OLE1, MeDIP, 2hr, 256X, rep2	3160436	GSM7762080	This study
	CDC43_1	CDC43, MeDIP, 2hr, 256X, rep1	5045882	GSM7762081	This study
	CDC43_2	CDC43, MeDIP, 2hr, 256X, rep2	4451495	GSM7762082	This study
	PCL10_1	PCL10, MeDIP, 2hr, 256X, rep1	1894009	GSM7762083	This study
	PCL10_2	PCL10, MeDIP, 2hr, 256X, rep2	4636618	GSM7762084	This study
	SHC1_1	SHC1, MeDIP, 2hr, 256X, rep1	1987853	GSM7762085	This study
	SHC1_2	SHC1, MeDIP, 2hr, 256X, rep2	1562438	GSM7762086	This study
	LSM4_1	LSM4, MeDIP, 2hr, 256X, rep1	3464136	GSM7762087	This study
	LSM4_2	LSM4, MeDIP, 2hr, 256X, rep2	4013404	GSM7762088	This study
	VHR1_1	VHR1, MeDIP, 2hr, 256X, rep1	2918360	GSM7762089	This study
	VHR1_2	VHR1, MeDIP, 2hr, 256X, rep2	2668382	GSM7762090	This study
	MBR1_1	MBR1, MeDIP, 2hr, 256X, rep1	3084859	GSM7762091	This study
	MBR1_2	MBR1, MeDIP, 2hr, 256X, rep2	2836143	GSM7762092	This study
	MNR2_1	MNR2, MeDIP, 2hr, 256X, rep1	3255820	GSM7762093	This study
	MNR2_2	MNR2, MeDIP, 2hr, 256X, rep2	3946345	GSM7762094	This study
	LHS1_1	LHS1, MeDIP, 2hr, 256X, rep1	2506307	GSM7762095	This study
	LHS1_2	LHS1, MeDIP, 2hr, 256X, rep2	3075927	GSM7762096	This study
	TRP2_1_minus	TRP2, -met, MeDIP, 2hr, 256X, rep1	2536546	GSM7762097	This study
	TRP2_2_minus	TRP2, -met, MeDIP, 2hr, 256X, rep2	2336606	GSM7762098	This study
	Ctrl_1_minus	Ctrl, -met, MeDIP, 2hr, 0X, rep1	3896569	GSM7762099	This study
	Ctrl_2_minus	Ctrl, -met, MeDIP, 2hr, 0X, rep2	3543876	GSM7762100	This study
	TRP2_1_plus	TRP2, +met, MeDIP, 2hr, 256X, rep1	3955143	GSM7762101	This study
	TRP2_2_plus	TRP2, +met, MeDIP, 2hr, 256X, rep2	4071011	GSM7762102	This study
	Ctrl_1_plus	Ctrl, +met, MeDIP, 2hr, 0X, rep1	3437007	GSM7762103	This study
	Ctrl_2_plus	Ctrl, +met, MeDIP, 2hr, 0X, rep2	3278012	GSM7762104	This study
	MATa_1_D_T	MATa, D, MeDIP, 2hr, 256X, rep1	1926066	GSM7762105	This study
	MATa_2_D_T	MATa, D, MeDIP, 2hr, 256X, rep2	1466199	GSM7762106	This study
	MATa_1_D_C	Ctrl_a, D, MeDIP, 2hr, 0X, rep1	2021677	GSM7762107	This study
	MATa_2_D_C	Ctrl_a, D, MeDIP, 2hr, 0X, rep2	1779710	GSM7762108	This study
	MATa_1_G_T	MATa, G, MeDIP, 2hr, 256X, rep1	2083163	GSM7762109	This study
	MATa_2_G_T	MATa, G, MeDIP, 2hr, 256X, rep2	1903572	GSM7762110	This study
	MATa_1_G_C	Ctrl_a, G, MeDIP, 2hr, 0X, rep1	3156921	GSM7762111	This study
	MATa_2_G_C	Ctrl_a, G, MeDIP, 2hr, 0X, rep2	2124145	GSM7762112	This study
	MATalpha_1_D_T	MATalpha, D, MeDIP, 2hr, 256X, rep1	1273791	GSM7762113	This study
	MATalpha_2_D_T	MATalpha, D, MeDIP, 2hr, 256X, rep2	1243979	GSM7762114	This study
	MATalpha_1_D_C	Ctrl_alpha, D, MeDIP, 2hr, 0X, rep1	1366041	GSM7762115	This study
	MATalpha_2_D_C	Ctrl_alpha, D, MeDIP, 2hr, 0X, rep2	1096130	GSM7762116	This study
	MATalpha_1_G_T	MATalpha, G, MeDIP, 2hr, 256X, rep1	1929813	GSM7762117	This study
	MATalpha_2_G_T	MATalpha, G, MeDIP, 2hr, 256X, rep2	1700940	GSM7762118	This study
	MATalpha_1_G_C	Ctrl_alpha, G, MeDIP, 2hr, 0X, rep1	858921	GSM7762119	This study
	MATalpha_2_G_C	Ctrl_alpha, G, MeDIP, 2hr, 0X, rep2	864864	GSM7762120	This study
	Ctrl_MLP1_MLP2_1	Ctrl, MLP1/2 mutant, MeDIP, 2hr, 0X, rep1	2443308	GSM8016823	This study
	Ctrl_MLP1_MLP2_2	Ctrl, MLP1/2 mutant, MeDIP, 2hr, 0X, rep2	1717788	GSM8016824	This study
	YBP2_MLP1_MLP2_1	YBP2, MLP1/2 mutant, MeDIP, 2hr, 256X, rep1	1583169	GSM8016825	This study
	YBP2_MLP1_MLP2_2	YBP2, MLP1/2 mutant, MeDIP, 2hr, 256X, rep2	1609459	GSM8016826	This study
M.CviPI-ID	Mlp1_ID_1	Mlp1_M.CviPI, MeDIP, rep1	1431200	GSM7762121	This study
	Mlp1_ID_2	Mlp1_M.CviPI, MeDIP, rep2	2112936	GSM7762122	This study
	Mlp1_Ctrl_1	M.CviPI, MeDIP, rep1	936321	GSM7762123	This study
	Mlp1_Ctrl_2	M.CviPI, MeDIP, rep2	2101152	GSM7762124	This study
Hi-C	HiC_1	HiC, -met, rep1	38432082	GSM7762058	This study
	HiC_2	HiC, -met, rep2	40855774	GSM7762059	This study
	HiC_Ctrl	HiC, Ctrl, -E2, rep1	44698489	GSM8016819	This study
	HiC_Ctrl_E2	HiC, Ctrl, +E2, rep1	36777542	GSM8016820	This study
	HiC_YBP2	HiC, YBP2, -E2, rep1	43994265	GSM8016821	This study
	HiC_YBP2_E2	HiC, YBP2, +E2, rep1	48372167	GSM8016822	This study
	HiC_+met	HiC, +met, rep1	NA	GSM5918358	Bastié, N. et al.
4C	4C_MATa_1	MATa, 4C, rep1	1886743	GSM7762050	This study
	4C_MATa_2	MATa, 4C, rep2	2680549	GSM7762051	This study
	4C_MATalpha_1	MATalpha, 4C, rep1	2756258	GSM7762052	This study
	4C_MATalpha_2	MATalpha, 4C, rep2	2642636	GSM7762053	This study
	4C_YBP2_1	YBP2, 4C, rep1	1601532	GSM7762054	This study
	4C_YBP2_2	YBP2, 4C, rep2	1830420	GSM7762055	This study
ChIP	MET4_IP_1	Met4, -met, ChIP, rep1	4718813	GSM7762056	This study
	MET4_IP_2	Met4, -met, ChIP, rep2	4898339	GSM7762057	This study
	Mlp1_IP_1	Mlp1-GFP, ChIP, rep1	NA	GSM4455453	Forey, R. et al.
	GFP_IP_1	GFP, ChIP, rep1	NA	GSM4455454	Forey, R. et al.
RNA-seq	RNAseq_1	RNA-seq, -met, rep1	33508576	GSM7762125	This study
	RNAseq_2	RNA-seq, -met, rep2	40154067	GSM7762126	This study
MicroC-XL	MicroC-XL_1	MicroC-XL, rep1	NA	GSM2262329	Hsieh, TS. et al.
	MicroC-XL_2	MicroC-XL, rep2	NA	GSM2262330	Hsieh, TS. et al.
	MicroC-XL_3	MicroC-XL, rep3	NA	GSM2262331	Hsieh, TS. et al.
