# Supplementary material for: DNA methylation-based high-resolution mapping of long-distance chromosomal interactions in nucleosome-depleted regions
Source: Nat Commun. 2024 May 22;15:4358. doi: 10.1038/s41467-024-48718-y (PMC11111806; doi:10.1038/s41467-024-48718-y)
Supplement: Supplementary file 3 — Description of Additional Supplementary Files [file 41467_2024_48718_MOESM3_ESM.pdf]

Description of Additional Supplementary Files

File name: Supplementary Data 1

Description: List of Nucleosome Depleted Regions (NDR) coordinates used in the DESeq2 analysis for differential methylation analysis.

File name: Supplementary Data 2

Description: Correlation matrix for heatmap presented in Figure 6B. We collected MTAC data for 487 interacting NDRs from 15 VPs located on 5 different chromosomes. In other words, each NDR has 15 MTAC signals (log2FC) associated with different VPs. The pair-wise Pearson's correlation of MTAC signals was calculated for each NDR with the rest of 486 NDRs. A 487 X 487 correlation coefficient matrix was generated and clustered hierarchically by Wald's method using the scipy.cluster.hierarchy package (version 1.12.0).

File name: Supplementary Data 3

Description: Information of viewpoints and captured chromosomal interactions. This data is a direct output of DESeq2 differential analysis. Aligned reads on each NDR were counted by Subread featureCounts (version 2.0.3) with the parameters "-p -O". The NDR coordinates (Supplementary Data 1) were generated by applying a previously described NDR annotation model to MNase-seq data in Kubik et al. Differential methylation analysis was performed on data from the targeted and control strains. Fold change and P-value were generated by DESeq2 (version 1.38.0) without outliers filtering. log2 (Fold Change) > 0.7 and the adjusted P-value < 0.05 were used as the cutoff for identifying differentially methylated regions.
