# Supplementary material for: DNA methylation-based high-resolution mapping of long-distance chromosomal interactions in nucleosome-depleted regions
Source: Nat Commun. 2024 May 22;15:4358. doi: 10.1038/s41467-024-48718-y (PMC11111806; doi:10.1038/s41467-024-48718-y)
Supplement: Supplementary file 7 — Reporting Summary [file 41467_2024_48718_MOESM7_ESM.pdf]

nature portfolio

Corresponding author(s): Lu Bai

Last updated by author(s): Apr 29, 2024

Reporting Summary

Nature Portfolio wishes to improve the reproducibility of the work that we publish. This form provides structure for consistency and transparency in reporting. For further information on Nature Portfolio policies, see our Editorial Policies and the Editorial Policy Checklist.

Statistics

For all statistical analyses, confirm that the following items are present in the figure legend, table legend, main text, or Methods section.

n/a	Confirmed	
☐	☒	The exact sample size (*n*) for each experimental group/condition, given as a discrete number and unit of measurement
☐	☒	A statement on whether measurements were taken from distinct samples or whether the same sample was measured repeatedly
☐	☒	The statistical test(s) used AND whether they are one- or two-sided<br>*Only common tests should be described solely by name; describe more complex techniques in the Methods section.*
☒	☐	A description of all covariates tested
☐	☒	A description of any assumptions or corrections, such as tests of normality and adjustment for multiple comparisons
☐	☒	A full description of the statistical parameters including central tendency (e.g. means) or other basic estimates (e.g. regression coefficient) AND variation (e.g. standard deviation) or associated estimates of uncertainty (e.g. confidence intervals)
☐	☒	For null hypothesis testing, the test statistic (e.g. *F*, *t*, *r*) with confidence intervals, effect sizes, degrees of freedom and *P* value noted<br>*Give P values as exact values whenever suitable.*
☒	☐	For Bayesian analysis, information on the choice of priors and Markov chain Monte Carlo settings
☒	☐	For hierarchical and complex designs, identification of the appropriate level for tests and full reporting of outcomes
☐	☒	Estimates of effect sizes (e.g. Cohen's *d*, Pearson's *r*), indicating how they were calculated

Our web collection on statistics for biologists contains articles on many of the points above.

Software and code

Policy information about availability of computer code

Data collection	MATLAB 2017b was used for imaging data collection.
Data analysis	fastp (version 0.23.2), BWA (version 0.7.17), and Picard MarkDuplicates (version 3.0.0) from Galaxy were used for processing the sequencing data. deeptools bamCoverage (version 3.5.1), Subread featureCounts (version 2.0.3), MACS2 (version 2.2.7.1), and DESeq2 (version 1.38.0) from Galaxy were used for sequencing data analysis. pipe4C and PeakC were used for 4C analysis. HiC-pro (version 3.1.0) was used for Hi-C analysis. SciPy (version 1.12.0) was used for correlation analysis. Seaborn (version 0.11.0) was used for heatmap plot. Custom scripts used for correlation analysis and heatmap in Figure 6 are available at github.com/yzl452/MTAC.

For manuscripts utilizing custom algorithms or software that are central to the research but not yet described in published literature, software must be made available to editors and reviewers. We strongly encourage code deposition in a community repository (e.g. GitHub). See the Nature Portfolio guidelines for submitting code & software for further information.

Data

Policy information about availability of data

All manuscripts must include a data availability statement. This statement should provide the following information, where applicable:

- Accession codes, unique identifiers, or web links for publicly available datasets
- A description of any restrictions on data availability
- For clinical datasets or third party data, please ensure that the statement adheres to our policy

The sequencing data generated in this study have been deposited into the Gene Expression Omnibus (GEO) database under accession code GSE242400. The viewpoint information and detected chromosomal interactions are listed on Supplementary Data 3. The summary of datasets generated or used in this study is listed on Supplementary Table 3. Source data are provided with this paper.

Research involving human participants, their data, or biological material

Policy information about studies with human participants or human data. See also policy information about sex, gender (identity/presentation), and sexual orientation and race, ethnicity and racism.

Reporting on sex and gender	n/a
Reporting on race, ethnicity, or other socially relevant groupings	n/a
Population characteristics	n/a
Recruitment	n/a
Ethics oversight	n/a

Note that full information on the approval of the study protocol must also be provided in the manuscript.

Field-specific reporting

Please select the one below that is the best fit for your research. If you are not sure, read the appropriate sections before making your selection.

☒ Life sciences ☐ Behavioural & social sciences ☐ Ecological, evolutionary & environmental sciences

For a reference copy of the document with all sections, see nature.com/documents/nr-reporting-summary-flat.pdf

Life sciences study design

All studies must disclose on these points even when the disclosure is negative.

Sample size	No statistical method was used to predetermine sample sizes. The sample size was determined based on common practices and similar studies. Two biological replicates per group were used for DNA and RNA sequencing analysis in this study. Two biological replicates and three technical replicates per group were used for qPCR analysis and other in vivo experiments in this study. All sample sizes were listed in the corresponding figures or figure legends.
Data exclusions	No data were excluded.
Replication	Replication numbers are defined in the figure legends. For DNA and RNA sequencing analysis, two biological replicates per group were used. For qPCR and other in vivo experiments, two biological replicates and three technical replicates per group were used. All attempts at replication were successful.
Randomization	For all experiments, yeast strains and conditions in each group were kept as closely as possible to each other to reduce any potential co-variates. Biologically independent yeast colonies were randomly selected after cloning for each treatment group.
Blinding	Blinding was not possible as experiments were often performed by a single investigator. However, quantifications were performed using computational pipeline and threshold applied equally to all conditions with no bias.

Reporting for specific materials, systems and methods

We require information from authors about some types of materials, experimental systems and methods used in many studies. Here, indicate whether each material, system or method listed is relevant to your study. If you are not sure if a list item applies to your research, read the appropriate section before selecting a response.

Materials & experimental systems

n/a	Involved in the study	
☐	☒	Antibodies
☒	☐	Eukaryotic cell lines
☒	☐	Palaeontology and archaeology
☒	☐	Animals and other organisms
☒	☐	Clinical data
☒	☐	Dual use research of concern
☒	☐	Plants

Methods

n/a	Involved in the study	
☐	☒	ChIP-seq
☒	☐	Flow cytometry
☒	☐	MRI-based neuroimaging

Antibodies

Antibodies used	1. 5-Methylcytosine Monoclonal Antibody (Thermo Fisher, 33D3)<br>2. TAP Tag Polyclonal Antibody (Thermo Fisher, CAB1001)<br>3. Anti-V5 tag antibody [SV5-Pk1] (Abcam, ab27671) (1:1000 dilution)<br>4. Anti-Actin antibody [mAbGEa] (Abcam, ab230169) (1:2000 dilution)<br>5. Anti-Mouse IgG (Fab specific) - Peroxidase antibody produced in goat (Sigma, A9917) (1:2000 dilution)
Validation	Validation statements, relevant citations, and antibody profiles are available from manufactures:<br>1. https://www.thermofisher.com/antibody/product/5-Methylcytosine-Antibody-clone-33D3-Monoclonal/MA5-38432<br>2. https://www.thermofisher.com/antibody/product/TAP-Tag-Antibody-Polyclonal/CAB1001<br>3. https://www.abcam.com/products/primary-antibodies/v5-tag-antibody-sv5-pk1-ab27671.html<br>4. https://www.abcam.com/products/primary-antibodies/actin-antibody-mabgea-ab230169.html<br>5. https://www.sigmaaldrich.com/US/en/product/sigma/a9917

Plants

Seed stocks	n/a
Novel plant genotypes	n/a
Authentication	n/a

ChIP-seq

Data deposition

☒ Confirm that both raw and final processed data have been deposited in a public database such as GEO.

☒ Confirm that you have deposited or provided access to graph files (e.g. BED files) for the called peaks.

Data access links<br>*May remain private before publication.*	https://www.ncbi.nlm.nih.gov/geo/query/acc.cgi?acc=GSE242395
Files in database submission	MET4_IP_1_R1_001.fastq.gz<br>MET4_IP_2_R1_001.fastq.gz<br>MET4_IP_1_R2_001.fastq.gz<br>MET4_IP_2_R2_001.fastq.gz<br>MET4_IP_1.bigwig<br>MET4_IP_2.bigwig
Genome browser session<br>(e.g. UCSC)	https://genome.ucsc.edu/s/yzl452/Met4_ChIP%2Dseq

Methodology

Replicates	2 biological replicates were used
Sequencing depth	10 million of 150bp paired-end reads were aligned to the S. cerevisiae genome, 6.5 million of reads were uniquely mapped.
Antibodies	TAP Tag Polyclonal Antibody (Thermo Fisher, CAB1001)
Peak calling parameters	Default parameters from Galaxy were used for BWA mapping and MACS2 peak calling.
Data quality	Two biological replicates were pooled for peak calling. 53 peaks are at FDR 5% and above 5-fold enrichment. 45 peaks were kept after manual inspection of peak quality.

Software

fastp (version 0.23.2), BWA (version 0.7.17), and Picard MarkDuplicates (version 3.0.0) from Galaxy were used for processing the sequencing data. deeptools bamCoverage (version 3.5.1) and MACS2 (version 2.2.7.1) from Galaxy were used for visualization and peak calling.
